# Metal-Insulator-Semiconductor Photodetectors

**DOI:** 10.3390/s101008797

**Published:** 2010-09-28

**Authors:** Chu-Hsuan Lin, Chee Wee Liu

**Affiliations:** 1 Institute of Opto-Electronic Engineering, National Dong Hwa University, Hualien 97401, Taiwan; E-Mail: chlin0109@mail.ndhu.edu.tw; 2 Department of Electrical Engineering, Graduate Institute of Electronics Engineering, and Graduate Institute of Photonics and Optoelectronics, National Taiwan University, Taipei 10617, Taiwan

**Keywords:** MIS, metal-insulator-semiconductor, photodetector

## Abstract

The major radiation of the Sun can be roughly divided into three regions: ultraviolet, visible, and infrared light. Detection in these three regions is important to human beings. The metal-insulator-semiconductor photodetector, with a simpler process than the pn-junction photodetector and a lower dark current than the MSM photodetector, has been developed for light detection in these three regions. Ideal UV photodetectors with high UV-to-visible rejection ratio could be demonstrated with III–V metal-insulator-semiconductor UV photodetectors. The visible-light detection and near-infrared optical communications have been implemented with Si and Ge metal-insulator-semiconductor photodetectors. For mid- and long-wavelength infrared detection, metal-insulator-semiconductor SiGe/Si quantum dot infrared photodetectors have been developed, and the detection spectrum covers atmospheric transmission windows.

## 1. Introduction

Photo-detection ranging from ultraviolet (UV) through the visible to infrared (IR) regions is closely related to human life. UV light is usually divided into three regions: UV-A, UV-B, and UV-C [1]. UV-C (10 nm–280 nm) light has the largest photon energy among the three UV regions, and hence, it is the most harmful to human beings. However, most of it is absorbed by the atmospheric ozone layer. UV-B (280 nm–320 nm) can partially pass through the atmospheric ozone layer, and can lead to skin cancer. UV-A (320 nm–400 nm) has the smallest photon energy and causes less harm for people. UV photodetectors can provide early warning signs for preventing overexposure. Detection of UV light can also be used in chemical and biological analysis. Visible light covers the range of wavelengths from 400 nm to 700 nm. Human eyes are natural visible photodetectors. In order to reproduce surrounding sights, visible photodetectors are significant. Beyond the visible wavelengths, a broad range of wavelengths constitute the infrared region. The detection of infrared can be roughly divided into two groups: for wavelengths shorter than 2 μm, known as the near-infrared region, the detection can be used for telecommunications and optical interconnects. For wavelengths longer than 2 μm, corresponding to the mid- and long-wavelength infrared, the detection can be used for imaging systems in astronomy, military, medicine, and other applications [2,3].

The common types of photodetectors include p-i-n, metal-semiconductor-metal (MSM), and metal-insulator-semiconductor (MIS) structures [4,5]. When an intrinsic semiconductor is sandwiched between a highly doped p-type layer and a highly doped n-layer, we have the basic configuration of a p-i-n type detector. A built-in electric-field will be formed inside the whole structure. A reversed bias could enlarge this electric field. When a target light with a suitable wavelength irradiates this structure, electron-hole pairs could be generated and separated by the electric field. Photo-detection can be achieved with such a mechanism. The MSM detector has a similar photo-detection principle, with the electric field distributed inside the semiconductor region. For the MIS detector, an insulator layer is inserted between the metal and semiconductor. A thin insulator layer in the MIS structure can allow a significant tunneling gate current, which could be utilized for photodetectors. A deep-depletion region formed in the semiconductor of the MIS detector can provide the electric field for separation of photo-generated electron-hole pairs. Compared to p-i-n detectors, MIS detectors offer a simpler process since the doping of PIN detectors is usually accomplished via implantation or diffusion. Since a thin insulator layer is between the metal and semiconductor, the dark currents of MIS detectors can usually be lower than those of MSM detectors. The extra surface barrier and decrease of interface states can contribute to the reduction of dark currents [6].

This review focuses on MIS photodetectors working in the UV, visible or infrared regions. First, the electrical characteristics of the MIS structure will be discussed. The MIS UV photodetectors using wide-bandgap semiconductor materials or metal filters will be discussed in Section 3. Then, the MIS structures for visible-to-near-IR detection employing the most broadly used semiconductor material, Si, will be discussed. Ge can be used to extend the cutoff wavelength of Si photodetectors. Finally, mid- and long-wavelength infrared detection by HgCdTe MIS photodetectors and MIS SiGe/Si quantum dot (well) infrared photodetectors will be introduced in Section 5.

## 2. Electrical Characteristics of MIS Structures

**Figure 1.**
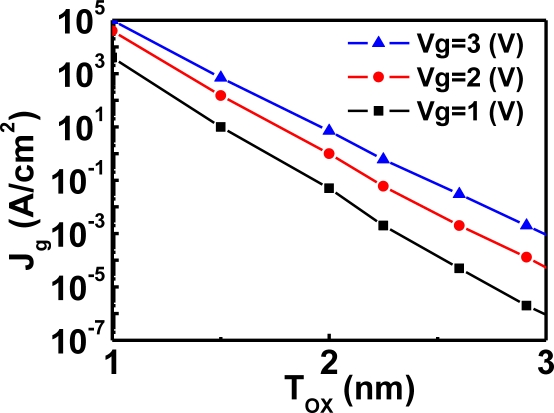
Gate tunneling current as a function of oxide thickness for various gate biases. In this example, the gate tunneling current is dominated by election tunneling from the conduction band of the vertical NMOS diode of an NMOSFET [7,8].

MOSFETs have become basic components of most electronic devices. In order to increase circuit performance, the quest to scale down MOSFETs continues. As the device size is scaled down, the oxide thickness decreases and the gate tunneling current along the vertical MOS structure (in the special case of MIS structures) then increases. Figure 1 shows the relation between the gate tunneling current and oxide thickness [7,8].

Figure 1 indicates that the direct tunneling current of the MOS structure with high-quality thermal oxide gradually becomes significant when the oxide thickness is less than 3 nm. For low-quality oxide, such as liquid-phase-deposition oxide, the current becomes significant even at a larger thickness with the help of trap-assisted tunneling.

As a photodetector, MOS diodes are usually operated at the inversion bias. For example, a positive bias should be applied on the Al/SiO_2_/p-Si diode for light detection. From [8], the dark current density *J* via the tunneling process of direct tunneling for the vertical MOS diode with an MOSFET transistor structure can be simplified as:

(1) J=A/ε_ox×M(V_ox)×V_g/T_ox×N(V_g)

with:

(2) $$M(V_{\mathrm{ox}})=\text{exp}(\frac{20}{\phi_{b}}{\left(\frac{|V_{\mathrm{ox}}|-\phi_{b}}{\phi_{b0}}+1\right)}^{\alpha }(1-\frac{\left|V_{\mathrm{ox}}\right|}{\phi_{b}}))\times \text{exp}[\frac{-B[1-{\left(1-|\frac{V_{\mathrm{ox}}}{\phi_{b}}|\right)}^{3/2}]T_{\mathrm{ox}}}{V_{\mathrm{ox}}}]$$

and at the inversion bias, the carrier density *N*(*V_g_*) can be approximated as:

(3) N(V_g)=ɛ_ox/T_ox [D⋅ln (1+exp ((V_g−V_th)/D))]

where:

*A*, *B*, *D*, and *α*: model parameters;
*T_ox_*: oxide thickness;
*V_ox_*: oxide voltage;
*φ_b0_*: semiconductor/oxide barrier height;
*φ_b_*: actual tunneling barrier height;
*V_th_*: threshold voltage.

**Figure 2.**
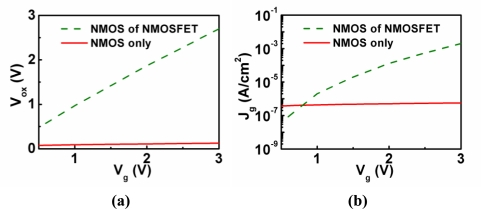
**(a)** The oxide voltage as a function of the gate voltage for a 3-nm-oxide NMOS with an NMOSFET transistor structure [8] and a 2.3 nm oxide NMOS without a transistor structure [9]; **(b)** Gate tunneling current density as a function of the gate voltage for a 2.9 nm oxide NMOS with an NMOSFET transistor structure [8] and a 2.3 nm oxide NMOS without a transistor structure [9].

**Figure 3.**
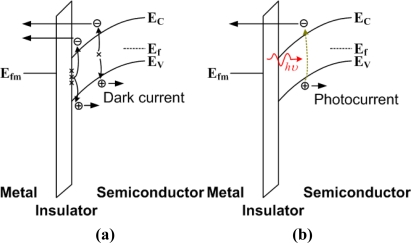
The current mechanisms of an MIS diode **(a)** without light illumination; **(b)** with light illumination. A positive bias (inversion bias) is applied for this metal/insulator/p-type semiconductor example.

Equation (2) shows that *M*(*V_ox_*) is a complicated function of *V_ox_*. The magnitude of *M*(*V_ox_*) will increase as *V_ox_* increases in the regions of interest. The “NMOS of NMOSFET” curve in Figure 2(a) indicates that *V_ox_* of an NMOS diode with a transistor structure is a linear function of the gate voltage (*V_g_*). In addition, the *V_g_*/*T_ox_* term and *N*(*V_g_*) term in Equation (1) also contribute to the increase of the direct tunneling current density *J* as *V_g_* increases. Overall, the direct tunneling current density of an NMOS diode with a transistor structure shows an exponential-like behavior as *V_g_* increases (“NMOS of NMOSFET” curve in Figure 2(b)). On the other hand, the NMOS diode without a transistor structure is quite different. At the inversion bias, inversion carriers cannot be supplied from the source and drain of an MOSFET transistor. This results in the soft pinning of *V_ox_* as *V_g_* increases (“NMOS only” curve in Figure 2(a)) [9]. In addition, the inversion carrier density will not be *V_g_* dependent as shown in Equation (3). Therefore, the dark direct tunneling current of an NMOS diode without a transistor structure is relatively constant in the log scale (“NMOS only” curve in Figure 2(b)). This dark inversion current density is dominated by the minority-carrier (electrons in p-Si, for example) generation rate at the oxide/semiconductor interface and in the deep-depletion region of the semiconductor (Figure 3(a)). For detector applications, an MIS diode without a transistor structure is preferred. Hence, the following discussion of MIS photodetectors will focus on those without transistor structures. Gate tunneling currents in MIS diodes without transistor structures have been comprehensively studied in [10].

**Figure 4.**
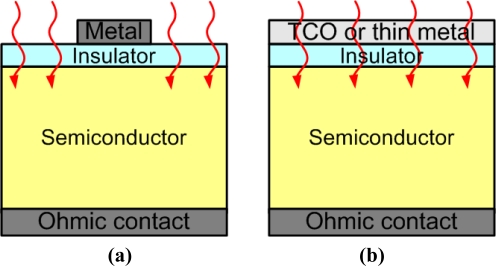
The schematic structures of metal-insulator-semiconductor photodetectors. **(a)** Light irradiates into the semiconductor from the unshadowed region when the metal is not transparent; **(b)** Light irradiates into the semiconductor passing through the transparent conducting oxide (TCO) or thin semi-transparent metal film.

Under light exposure at the inversion bias, excess electron-hole pairs are generated in the semiconductor and contribute to the photocurrent (Figure 3(b)). For the MIS photodetector with a nontransparent metal gate, the incident light is incorporated into the semiconductor from the edge of the metal gate (Figure 4(a)). In order to increase the responsivity of a photodetector, a transparent gate could be utilized, and the incident light would be incorporated into the semiconductor directly from the gate (Figure 4(b)). Transparent gates can be roughly classified into two categories. The first are the transparent conducting oxides (TCOs), such as indium tin oxide (ITO) or zinc oxide (ZnO). The other are the metal films with thicknesses of ∼10 nm [11]. Since the metal film is so thin, absorption of the incident light in the metal is suppressed.

The thickness of the insulator layer will influence both the dark currents and photocurrents of the MIS photodetectors. A thicker insulator results in smaller currents. For example, Monroy *et al.* have discussed the effects of the SiO_2_ thickness on GaN MIS photodetectors [12]. When the thicknesses of SiO_2_ was varied from 2.5 nm to 5 nm, the corresponding dark current densities (at −2 V) varied from ∼7 × 10^−5^ to 4 × 10^−6^ A/cm^2^, and the corresponding photocurrents (at 100 W) varied from ∼0.7 to 0.09 A, respectively. Meanwhile, the dark current density and photocurrent of a Schottky barrier GaN photodiode were ∼8 × 10^−4^ A/cm^2^ and 5 A. The thicker insulator could be used for lower noise operation, but the trade-off is the lower responsivity.

The speed of a photodetector may be limited by the carrier transit time or RC delay. If the photodetector is transit time limited, the bandwidth is given by [13]:

(4) $$f_{\text{transit}}∼\frac{2.4}{2\pi (W/v_{s}}∼\frac{0.4v_{s}}{W}$$

where *W* is the width of the depletion region, and *v_s_* is the saturation velocity. The term *v_s_* is a material-dependent parameter. In the deep-depletion situation, the *W* of an MIS diode can be approximated as [14]:

(5) $$W∼\sqrt{\frac{2{ɛ}_{\text{semiconductor}}\;V_{g}}{\text{eN}}}$$

where *N* is the doping concentration of the semiconductor. With Equations (4) and (5), the transit time limited bandwidth can be determined. If the photodetector is RC delay limited, the bandwidth will be [15]:

(6) $$f_{\text{RC}}=\frac{1}{2\pi \text{RC}}$$

where *R* includes the series resistance and load resistance. The *C* of an MIS photodetector is dominated by the semiconductor depletion capacitance in series with the insulator capacitance [16]. When the order of *f_transit_* is close to that of *f_RC_*, the resultant f should be [15]:

(7) $$f=\sqrt{\frac{1}{{1/f}_{\text{transit}}^{2}+{1/f}_{\text{RC}}^{2}}}$$

## 3. MIS UV Photodetectors

Applications of UV detection include chemical monitoring, biological sensing, missile plume detection, exposure monitoring, *etc.* The wide-bandgap semiconductor is suitable for UV detection when visible light will not contribute to the absorption [17]. The wide-bandgap III–V nitride, SiC, and Zn-based semiconductor MIS UV photodetectors will be discussed. Si, which has a much smaller bandgap than the III–V nitride semiconductor, is a strong absorber for UV light. A Si MIS structure with an appropriate selection of the gate electrode can also be used for UV detection.

### 3.1. III–V MIS UV Photodetectors

The III–V nitride semiconductors, (Al, In, Ga)N, are direct bandgap materials. The absorption edge of AlN is 200 nm, and the absorption edge of GaN is 365 nm [18]. Using (In, Ga)N alloys, the absorption edge can be extended to 400 nm. With varying Al mole fraction in (Al, In, Ga)N alloys, the detection edge can be tuned. These III–V nitride materials can be fabricated into simple MIS photodetectors. Compared with metal-semiconductor (Schottky barrier) photodiodes, insertion of a thin SiO_2_ layer for the III–V nitride photodetector can effectively decrease the dark current. For example, Monroy *et al*. have demonstrated the dark current reduction with the Au/SiO_2_/GaN MIS photodetector [16]. The magnitude of the dark current of the Au/SiO_2_/GaN MIS photodetector is four orders lower than the Au/GaN Schottky photodetector, and the noise spectral density of the MIS photodetector is also three orders lower than the Schottky photodetector due to the passivation by SiO_2_

Since GaN is a well-known III–V nitride material, the study on the GaN MIS UV photodetector will be very representative. Two GaN MIS UV photodetectors based on different techniques of insulator formation by different groups will be introduced here, and the results show that similar performances could be achieved. (I) A GaN MIS UV photodetector with a photo chemical-vapor-deposition (photo-CVD) SiO_2_ layer was studied by Chiou *et al.* [19]. The deuterium (D_2_) lamp was selected as a photo-CVD photo excitation source. The D_2_ lamp could effectively decompose reacting gas, and it resulted in a high quality SiO_2_ layer. The SiO_2_ film was 50 nm thick. The high frequency capacitance-voltage measurement showed that the D_it_ between SiO_2_ and GaN could be as low as 8.4 × 10^11^ cm^−2^ eV^−1^. Such a low D_it_ was believed to be due to effective decomposition by the D_2_ lamp. The low D_it_ was proved to lead to a low leakage current. As compared with the photodiode without the SiO_2_ film, the dark current was decreased by seven orders while the photocurrent was only decreased by four orders. Hence, the final photocurrent to dark current ratio was increased by three orders. The normalized detectivity (D^*^) was defined as [20]:

(8) $$D^{*}=\frac{\sqrt{A\Delta f}}{\mathrm{NEP}}=\frac{\sqrt{A\Delta f}}{i_{n}/R}$$

where *A* is the area of the investigated photodetector, and Δ*f* is the bandwidth of the photodetector. The noise equivalent power (NEP) was formulated as *i_n_/R*, where *i_n_* is the current noise and *R* is the responsivity. The difference between the photocurrent and dark current was divided by the input power to obtain the responsivity. The responsivity of a photodetector should be as large as possible. This ITO/photo-SiO_2_/GaN had a responsivity of ∼120 mA/W. The NEP was estimated to be 2.2 × 10^−9^ W. With a detector size of 200 × 200 μm^2^ and given bandwidth of 500 Hz, the normalized detectivity was calculated using Equation (8) to be 2.0 × 10^8^ cm Hz^0.5^W^−1^.

(II) Hwang *et al*. also demonstrated a GaN MIS UV photodetector with a liquid-phase-deposition (LPD) SiO_2_ layer [21]. LPD is a low-temperature (30 °C–40 °C) process, which could prevent the redistribution of concentrations at a high temperature. The deposited SiO_2_ could be annealed at 800 °C in order to achieve a denser film. The minimum D_it_ of this LPD-SiO_2_ device was the same (8.4 × 10^11^ cm^−2^ eV^−1^) as the photo-SiO_2_ device just mentioned. The responsivity of the 10 nm thick LPD-SiO_2_ MIS photodetector at the 366 nm wavelength was 112 mA/W, which was also close to the result of the photo-SiO_2_ photodetector. It should be noted that since the oxide was 10 nm thick, carriers could hardly tunnel through the oxide layer via the mechanism of direct tunneling. Hwang *et al*. indicated that defect-assisted tunneling might help the carrier transport. Although the normalized detectivity of this Al/LPD-SiO_2_/GaN MIS UV photodetector was not given, its detectivity could be estimated as follows: as discussed in [20], the *i_n_* (V ≠ 0) could be approximated as the shot noise (2eI_d_Δf)^1/2^, where *I_d_* was the dark current of the photodetector. Therefore, D^*;^ in Equation (8) could be simplified as:

(9) $$D^{*}=\frac{\sqrt{A}R}{\sqrt{{2\mathrm{eI}}_{d}}}$$

In Equation (9), the *A*/*I_d_* term corresponds to the reciprocal of the dark current density. It could be found that the dark inversion (depletion) current densities of ITO/photo-SiO_2_/GaN in [19] and Al/LPD-SiO_2_/GaN in [21] were of the same order of magnitude. Since the responsivities of both devices are close, the normalized detectivities would be at the same order, too.

**Figure 5.**
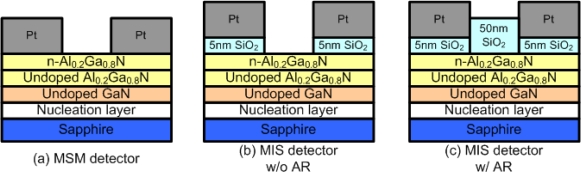
Three different AlGaN/GaN structures: **(a)** MSM detector; **(b)** MIS detector without anti-reflection coating and **(c)** MIS detector with anti-reflection coating. The 50 nm thick SiO_2_ layer in (c) acts as both a passivation layer and anti-reflection coating. Reproduced from [23] with permission of Elsevier (2006).

The most common source of UV light is the Sun, which also radiates visible light. In order to indicate the UV intensity, the development of visible-blind UV photodetectors is important [22]. The characteristics of three different structures (Figure 5) of AlGaN/GaN photodetectors have been compared, and the MIS photodetector was found to have a higher UV-to-visible rejection ratio as compared with the MSM photodetector [23].

**Figure 6.**
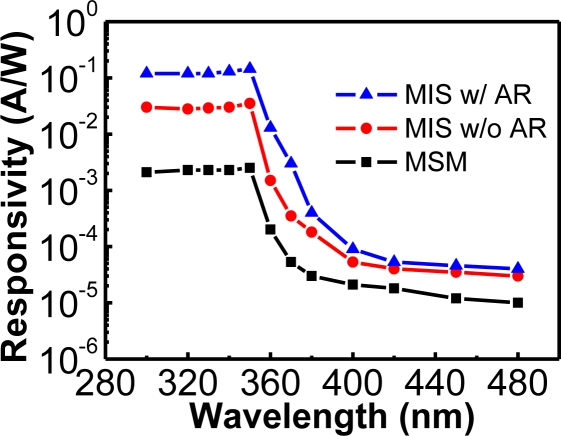
Spectral responsivities of these three different photodetectors shown in Figure 5. The AlGaN/GaN MIS photodetector with AR coating has the highest UV-to-visible rejection ratio. Reproduced from [23] with permission of Elsevier (2006).

First, on the AlGaN/GaN sample, two inter-digitated Pt contacts were deposited to form the MSM photodetector (Figure 5(a)). If a 5 nm thick photo-CVD SiO_2_ layer could be deposited before the deposition of Pt gates, an un-optimized MIS photodetector was able to be formed (Figure 5(b)). Another 50 nm thick SiO_2_ deposited between two inter-digitated contacts achieved the optimized MIS photodetector with anti-reflection (AR) coating (Figure 5(c)). Due to the passivation of the 50 nm thick SiO_2_ layer in the MIS photodetector with AR coating, the reduced surface states resulted in the lowest dark current among these three photodetectors. On the other hand, the photocurrent of the MIS photodetector with AR coating was larger than the MIS photodetector without AR coating since the reflection of the incident light could be suppressed. In addition, the passivation of 50 nm thick SiO_2_ could also reduce the recombination of photo-generated carriers. The resultant spectral responsivities of these three photodetectors were shown in Figure 6. The UV-to-visible rejection ratio of the MIS photodetector with AR coating was the largest, and the ratio was more than three orders of magnitude.

Some typical III–V nitride based UV photodetectors with different configurations are compared in Table 1. The dark current densities are shown for reference. However, as discussed in [26], the uncertainty of the junction area makes the direct comparison of current densities confusing. If the compared devices are fabricated into similar structures, the comparison would be much meaningful. For example, MIS and MSM AlGaN/GaN photodetectors in [23] show that the insertion of an insulator layer indeed reduces the dark current densities. From this table, we can still conclude that the p-i-n type photodetectors have the potential to achieve the lowest dark current densities.

### 3.2. SiC MIS UV Photodetectors

**Figure 7.**
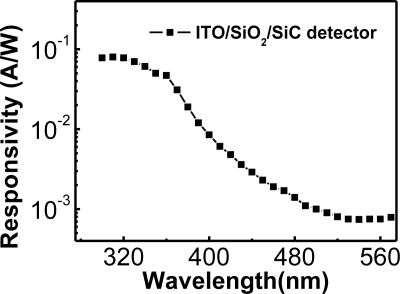
Spectral responsivity of the ITO/SiO_2_/SiC MIS photodetector. The UV (310 nm)-to-visible (500 nm) rejection ratio was ∼80. Reproduced from [31] with permission of Elsevier (2003).

SiC is also a wide-bandgap material, which can be used for UV photo-detection. The SiC pn junction photodiodes and Schottky barrier (SB) photodiodes have been well developed [30]. A 4H-SiC MIS photodetector with a photo-CVD SiO_2_ layer was also fabricated [31]. The D_it_ between SiC and photo-CVD SiO_2_ deposited at 150, 300, and 500 °C was 3.3 × 10^12^, 3.2 × 10^12^, and 5.7 × 10^11^ cm^−2^ eV^−1^, respectively. The higher temperature was able to provide more thermal energy for photo-decomposition of SiH_4_ and O_2_ gases, which resulted in a better SiO_2_/SiC interface. The surface morphology of a bare SiC substrate and a substrate with a photo-CVD SiO_2_ layer were studied by atomic force microscopy (AFM). The AFM showed that the root-mean-square roughness of surfaces without and with SiO_2_ layers were 0.32 and 4.24 nm, respectively. Although the substrate with an SiO_2_ layer had a rougher surface, its electrical characteristics do not reveal obvious disadvantages. The ITO/SiO_2_/SiC MIS photodetector was compared with an ITO/SiC SB photodetector. At 10 V, the dark current densities of MIS and SB detectors were 1.3 × 10^−6^ and 1.1 × 10^−3^ A/cm^2^, respectively. Both the dark current and photocurrent of the ITO/SiO_2_/SiC MIS detector were smaller than those of the ITO/SiC SB detector. Overall, the photocurrent to dark current ratio of the ITO/SiO_2_/SiC MIS detector was still much larger than that of the ITO/SiC SB detector. The spectral responsivity of the ITO/SiO_2_/SiC MIS detector was studied as shown in Figure 7. The UV-to-visible rejection ratio was ∼80, which was much lower than that of AlGaN/GaN MIS detector with AR coating as discussed in the previous subsection. A high performance 4H-SiC p-i-n UV photodetector was demonstrated by Chen *et al.* with a UV-to-visible rejection ratio larger than 10^3^ [32]. Much effort should be done to improve the rejection ratio of MIS detectors to compete with p-i-n ones.

### 3.3. Zn-Based MIS UV Photodetectors

Some II–VI semiconductor, like ZnSe, is a promising wide-bandgap material, too. The bandgap of ZnSe is about 2.6 eV [33]. ZnSe MIS photodetectors with different insulator layers have been investigated, including SiO_2_ and Ba_0.25_Sr_0.75_TiO_3_ (BST) [34]. With the insertion of an SiO_2_ layer or a BST layer, the dark currents decreased more than one order of magnitude as compared with ZnSe SB photodetectors. The UV (400 nm)-to-visible (500 nm) rejection ratio were about 120, 50, and 10 for ITO/SiO_2_/ZnSe, ITO/BST/ZnSe, and ITO/ZnSe photodetectors, respectively. The corresponding detectivities were 2.55 × 10^12^, 1.67 × 10^12^, and 1.5 × 10^11^ cmHz^0.5^W^−1^. The MIS structure was desirable for ZnSe UV photodetectors.

The superiority of the MIS structure over the MSM structure has also been shown for the material of ZnO, which has the bandgap of 3.37 eV [35]. For ZnO, the shortcomings of a pn-junction-type photodetector include not only the process complexity, but also the difficulty on formation of p-type ZnO [36]. The reproducibility and reliability of ZnO p-i-n photodetectors will be the issues. Hence, an MIS structure of ZnO is very favorable for photodetector applications. The test structures were very similar to the MSM detector and MIS detector without AR coating shown in Figure 5, and the probably optimized structure of the MIS detector with AR coating was not yet presented. Nevertheless, the high UV (370 nm)-to-visible (450 nm) rejection ratio of 3.8 × 10^3^ of this ZnO MIS photodetector was still larger than that of 2.4 × 10^2^ of the ZnO MSM photodetector. The quite low long-wavelength responsivity of this MIS detector contributed to its excellent rejection ratio.

**Figure 8.**
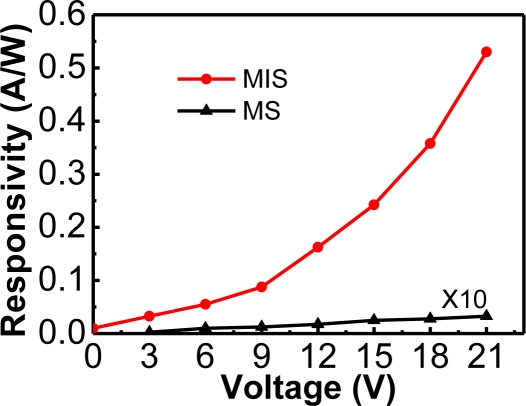
Responsivity as a function of applied voltage for the Au/MgO/MgZnO MIS and Au/MgZnO MS photodetectors. The responsivity of the MIS detector increased exponentially, meanwhile the MS detector showed a linear behavior. Reproduced from [37] with permission of the American Chemical Society (2010).

Furthermore, a derivative of ZnO, MgZnO, was fabricated into an Au/MgO/MgZnO MIS photodetector [37]. Such an Au/MgO/MgZnO photodetector possessed a high internal gain, and hence it is worthy of mention here. The MgZnO semiconductor film was deposited on the sapphire substrate by the metal-organic chemical vapor deposition technique. Subsequently, an MgO insulator layer was deposited by the sputtering technique. Finally, a 20 nm thick Au gate on the MgO layer and a ring-shaped Al contact on the MgZnO were deposited to form the MIS photodetector. For comparison, a metal-semiconductor photodetector without the MgO insulator film was also prepared. The responsivity *versus* applied voltage curves displayed interesting behaviors (Figure 8). The responsivity of the MIS photodetector increased exponentially with the increasing bias, meanwhile the MS photodetector showed a typical linear behavior [38,39].

**Figure 9.**
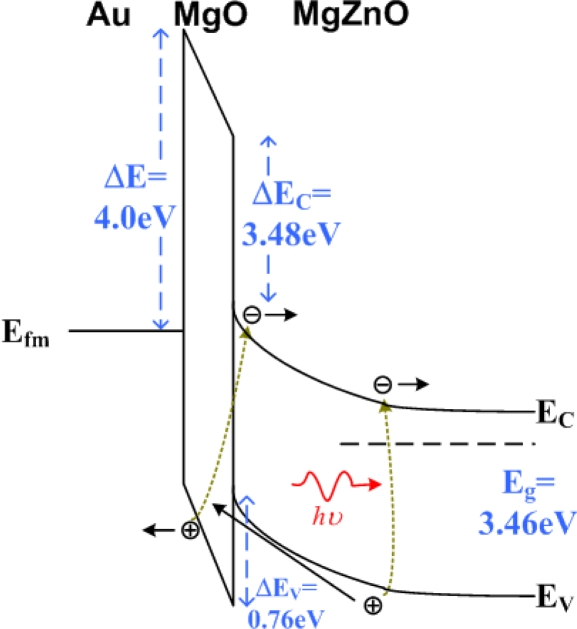
The schematic mechanism of optical gain in the MIS photodetector. With the multiplication process, the external quantum efficiency of the MIS detector could achieve 200% at 21 V [37].

The estimated external quantum efficiencies of the MIS and MS photodetectors were 22% and 0.26% at 5 V, respectively. The external quantum efficiency of the MIS photodetector increased to 200% at 21 V. The excellent results of the MIS photodetector came from the optical gain through the impact ionization process. Figure 9 shows the mechanism of optical gain in the MIS photodetector. When the UV light irradiated to the MgZnO semiconductor, electron-hole pairs could be generated. Photo-generated holes drifted toward the Au contact through the MgO insulator layer. These holes gained much kinetic energy in the trip, and extra electron-hole pairs were then generated via the impact ionization process. This multiplication process contributed to the exponential increase on the responsivity as a function of the applied voltage.

### 3.4. Metal-Filter Si MIS UV Photodetectors

**Figure 10.**
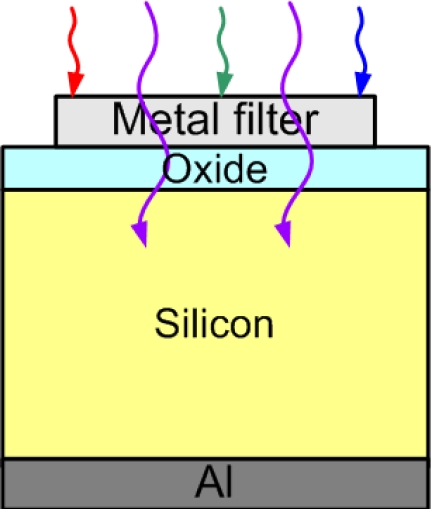
The schematic structure of a metal-filter (such as Ag) Si MIS UV photodetector. Incident photons were firstly selected by the gate, and then the remaining photons might be absorbed by Si to form the photocurrent.

Si is a strong absorber of UV light. For example, the absorption length at the 320 nm wavelength is below 10 nm in Si. The typical Si UV photodetectors are classified into two categories [30]. One are the diffused photodiodes with depletion regions between the p and n layers. The other is the SB photodiodes with depletion regions at the metal/semiconductor interfaces. Applications of UV detection based on Si are usually limited since Si has a broadband absorption for the solar spectrum. A visible-blind design for the Si UV photodetector is desirable. Ho *et al.* have demonstrated a Si MIS UV photodetector using Ag as the filter [40]. A ∼3 nm thick SiO_2_ layer was grown on an n-type Si wafer by rapid thermal oxidation. After oxidation, the Ag gate was deposited for band selection of UV light. Al was then evaporated on the backside of the Si wafer to form an Ohmic contact. The schematic structure of the Si MIS UV photodetector is shown in Figure 10. Incident photons were firstly selected by the Ag gate, and then the remaining photons might be absorbed by Si to generate electron-hole pairs. These photo-generated electron-hole pairs in Si were separated by the electric field in the depletion region. Since the absorption length of the UV light in Si was very short, the depletion region should be as close to the incident surface as possible. As compared to the p-i-n structure, the MIS structure could have the depletion region closer to the surface. In principle, the MIS photodetector would achieve the higher quantum efficiency [41].

**Figure 11.**
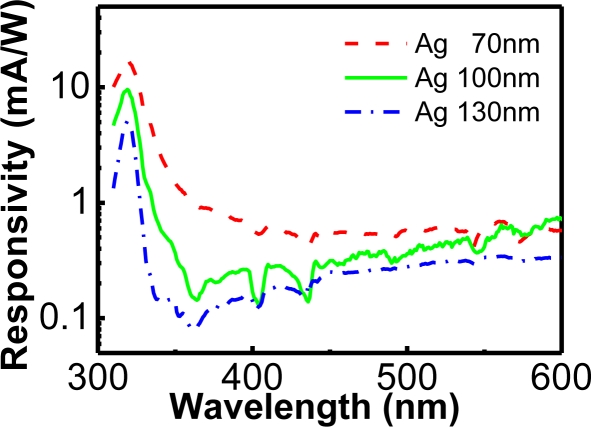
Spectral responsivities of the Ag/SiO_2_/Si photodetectors with different thicknesses of Ag. The transmission of UV band at the ∼319 nm wavelength was due to the material property of Ag [40].

When the thicknesses of Ag gates were varied from 70 to 130 nm, the peaks of the spectral responsivities were still located at the wavelength of 319 nm, with values of 17.3 to 5.1 mA/W (Figure 11). The transmission of UV band at the ∼319 nm wavelength was due to the material property of Ag (spectral dependence of absorption and reflection). The full width at half magnitude (FWHM) decreased as the thickness of Ag increased too. The FWHM decreased from 17 to 11 nm when the thickness of Ag was varied from 70 to 130 nm. Although the UV-to-visible rejection ratio of the metal-filter Si MIS photodetector was smaller than the AlGaN/GaN MIS photodetector mentioned in Subsection 3.1, a smaller FWHM could be achieved with such a photodetector.

## 4. MIS Visible-to-Near-IR Photodetectors

The spectral distribution of terrestrial solar radiation shows that the visible band has the largest intensity. Creatures, including human beings, on the surface of Earth, are the most sensitive to the visible light, therefore, the development of visible photodetectors is important. Si is the most promising material for visible detection since it has a suitable bandgap (1.12 eV) for visible-light absorption and it is one of the most abundant materials on Earth. Si can also detect the useful near infrared at the 850 nm wavelength. Near infrared has many significant applications, such as optical interconnects and optical communications [42]. In order to extend the detection wavelength to the other useful near-infrared bands of ∼1.3 and 1.55 μm, Ge, which is compatible with the mature Si process, is utilized. Representative MIS structures for visible or near-infrared photodetectors are introduced in this section.

### 4.1. Si-Based MIS Visible-to-Near-IR Photodetectors

Si-based MIS photodetectors are desirable due to the probability of integration with ULSI circuits. The photocapacitive mode MIS photodetector was proposed in 1980 [43], and optical sensors based on transient behaviors in MIS-capacitors were discussed by Malik *et al.* in 2004 [44,45]. However, MIS photodetectors using tunneling-photocurrent mode are more broadly used [46–49]. First, the simple NMOS and PMOS Si tunnel diodes will be introduced. The ultrathin (2.3 and 2.7 nm) oxide layers were grown by rapid thermal oxidation on the 1–5 cm p-type and n-type Si wafers in order to form the NMOS and PMOS tunneling diodes, respectively [9,50].

**Figure 12.**
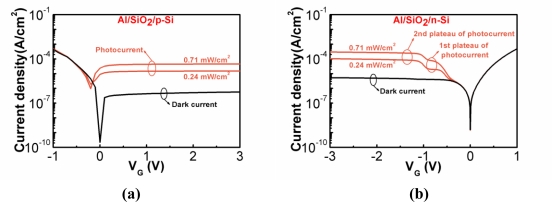
Dark current and photocurrents of the **(a)** Al/SiO_2_/p-Si MIS (NMOS) diode; **(b)** Al/SiO_2_/n-Si MIS (PMOS) diode. The inversion biases were positive and negative voltages for NMOS and PMOS photodetectors, respectively [9,50].

The J-V curves of the NMOS and PMOS photodetectors are shown in Figures 12(a) and 12(b), respectively. A metal halide lamp was used to excite the photocurrents. The dark current and photocurrents of the NMOS photodetector remained almost constant in the log scale when the positive bias was applied. Thermally generated electron-hole pairs at the SiO_2_/p-Si interface and in the depletion region in p-Si contributed to the dark current. With light exposure, the generation rate of electron-hole pairs increased and thus the gate tunneling current increased. For such ultrathin SiO_2_, the tunneling rate here was not a limiting factor for electrons tunneling from the p-Si to the Al gate, and hence these currents could keep almost constant with increased biases. Figure 12 shows the photocurrents under different light intensity. If ITO was used instead of Al as the gate electrode, the magnitude of the photocurrent could be increased by a factor of 20 [9].

**Figure 13.**
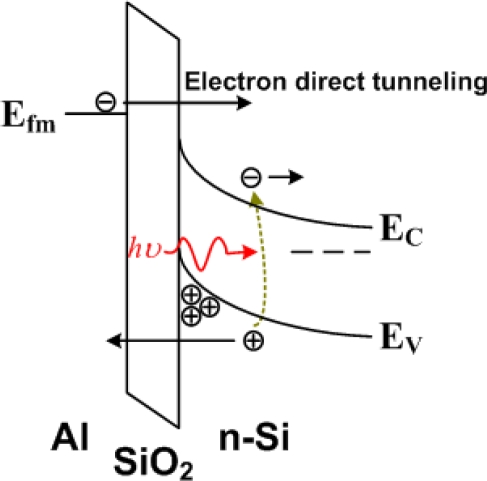
The schematic diagram of an Al/SiO_2_/n-Si MIS (PMOS) diode. The photo-generated holes contributed to the first plateau of the photocurrent and the electron direct tunneling contributed to the second plateau [50].

The mechanism of the photocurrent in the PMOS photodetector was a little more complicated. A kink appeared in the photocurrents but not in the dark current. The first plateau of the photocurrent was also due to the excess electron-hole-pair generation with light exposure. The second plateau was due to the electron direct tunneling from the Al gate to n-Si (Figure 13). The hole concentration at the SiO_2_/n-Si interface was determined by the tunneling rate and photo-generation rate of holes. As the negative bias continuously increased, hole concentration at the interface increased, and the oxide voltage increased slightly. The Fermi level of Al would arise away from the level of the conduction band edge of n-Si at the SiO_2_/n-Si interface, and the electron direct tunneling from the Al gate to n-Si occurred. Hence, there was a second plateau of the photocurrent. A similar phenomenon was observed by Hashimoto *et al.* [51].

**Figure 14.**
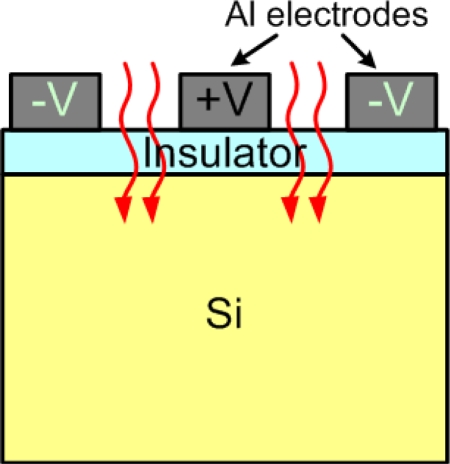
The schematic structure of a metal-insulator-semiconductor-insulator-metal photodetector. Both the anode and cathode electrodes were on the thin-insulator side.

In the above design, the gate electrodes and back Ohmic contacts were on different sides of the Si substrates. Both electrodes could be formed on the same side of thin-oxide/Si substrates to fabricate interdigitated metal-insulator-semiconductor-insulator-metal (MISIM) photodetectors (Figure 14) [45,52]. Such a structure could be compared with the MSM structure with the same metal pattern, and then the advantages of the insertion of an insulator layer became obvious. Seto *et al.* showed that the insertion of a thin SiO_2_ layer effectively reduced the dark current by a factor of 5.2 [52]. The increase in the dark current with the increasing bias was weaker. Furthermore, the responsivity was increased by a factor of 3.5 with such an MISIM structure. The responsivity measurement was operated under 635 nm laser diode illumination. At 5 V, the responsivities of the MISIM and MSM photodetectors were 0.39 and 0.11 A/W, respectively. In addition, the internal quantum efficiency of the MISIM detector was shown to be 1.8 larger than 0.5 of the MSM detector. There might be holes trapped at the insulator/semiconductor interface. These holes induced an additional electron tunneling current, which made the internal quantum efficiency of the MISIM detector larger than unity.

Due to the Si bandgap, the near infrared at the optical communication wavelengths of 1,300 and 1,550 nm cannot be detected. The detection performance at the wavelength of 820 nm is also limited. Five periods of SiGe quantum-dot layers were incorporated into the Si substrate to improve the near-infrared performance of MIS photodetectors [53]. The SiGe/Si quantum dots were grown on the Si substrate by the ultrahigh vacuum chemical vapor deposition with the Stranski-Krastanov growth mode. The SiGe layer acted as a low-bandgap region, which enhanced the absorption coefficient and extended the cut-off wavelength as compared with the Si device. The responsivities of the MIS SiGe/Si quantum-dot photodetector at wavelengths of 820, 1,300, and 1,550 nm were 130, 0.16, and 0.08 mA/W, respectively. If the number of periods of SiGe quantum-dot layers was increased to 20, a responsivity of 600 mA/W could be achieved at the wavelength of 850 nm.

### 4.2. Bulk Ge MIS Visible-to-Near-IR Photodetectors

**Figure 15.**
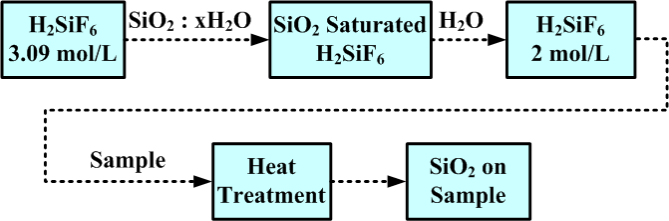
The process flow of liquid phase deposition. The process can be operated at the temperature below 100 °C, and a thin (∼2 nm) SiO_2_ layer can be deposited on the Ge substrate [53].

As mentioned in the previous subsection, Ge can be used for detection of near infrared at the 1,300 and 1,550 nm wavelengths. Ge photocapacitive mode MIS infrared photodetectors were proposed in 1979 [54]. In order to demonstrate the tunneling-photocurrent mode Ge MIS photodetector, a thin SiO_2_ layer was formed on the Ge substrate with the process of low-temperature liquid phase deposition (LPD) [55]. Note that the insulator layer was SiO_2_ instead of GeO_2_ since GeO_2_ was unstable and had a worse quality. The detailed LPD process flow is shown in Figure 15 [53].

**Figure 16.**
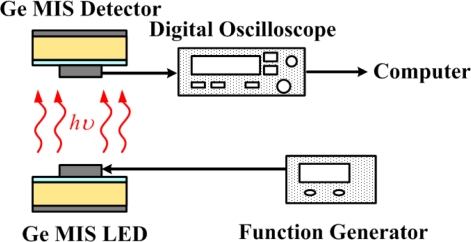
Data communication system using a Ge MIS photodetector and a Ge MIS LED. The same MIS structure was used with different biases for different applications. The Ge MIS structure with an inversion bias acted as a detector, while it acted as an LED with an accumulation bias [55].

In [55], Ge MIS structures were both used as photodetectors and light-emitting diodes (LEDs). The Ge MIS structure with an inversion bias acted as a photodetector, while it acted as an LED with an accumulation bias. The responsivities of the bulk Ge MIS photodetector at wavelengths of 1,310 and 1,550 nm were 180 and 53 mA/W, respectively. The data communication in free space has been demonstrated using a Ge MIS photodetector and a Ge MIS LED (Figure 16).

**Figure 17.**
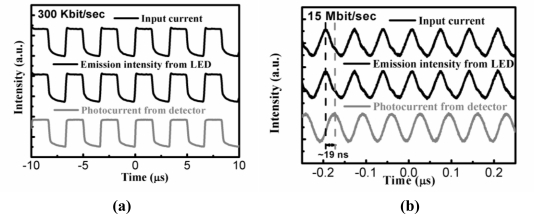
Modulation results of the data communication using a Ge MIS photodetector and a Ge MIS LED with a frequency of **(a)** 300 Kbit/sec; **(b)** 15 Mbit/sec. A delay time of ∼19 ns was observed in (b). Reprinted from [55] with permission of the American Institute of Physics (Copyright 2008).

The function generator was used to modulate the Ge MIS LED. Then, emission from the Ge MIS LED was detected by the Ge MIS photodetector. Finally, the corresponding photocurrent was read from the output instrument. The results of the data communication of this system are shown in Figure 17. When the modulation frequency is 300 Kbit/s, both the light intensity of the Ge MIS LED and the photocurrent of the Ge MIS photodetector followed the modulated input current. When the modulation frequency was as high as 15 Mbit/s, a delay time of ∼19 ns was observed between the light intensity of the Ge MIS LED and the photocurrent of the Ge MIS photodetector. The bandwidth of this Ge MIS photodetector was estimated to be ∼300 MHz. Hence, the modulation frequency for this free space data communication was limited by this Ge MIS photodetector. Optimized structures with, such as a small active area or a high-quality oxide layer without trapping would be needed to increase the modulation frequency.

**Figure 18.**
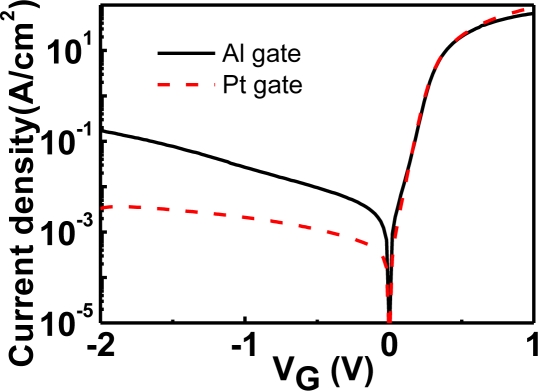
The dark currents of Al/SiO_2_/n-Ge and Pt/SiO_2_/n-Ge photodetectors. The dark inversion current of the Pt gate device was smaller due to the suppression of electron tunneling from the metal to n-Ge [56].

A suitable selection of the metal in the metal/SiO_2_/n-Ge photodetector may reduce the dark current which then improves the signal-to-noise ratio. In Figure 13, a mechanism of electron direct tunneling under light exposure was studied. Such an electron direct tunneling also contributed to the dark current. If the high-work-function metal was used, the dark current could be decreased with the suppression of electron tunneling from the metal to n-Ge. Dark inversion current reduction has been achieved with the high-work-function metal, Pt (5.65 eV), instead of the typical low-work-function metal, Al (4.1 eV) for Ge MIS photodetectors (Figure 18) [56].

### 4.3. Thin-Film Ge MIS Visible-to-Near-IR Photodetectors

**Figure 19.**
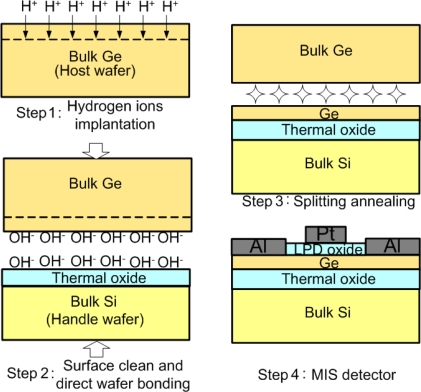
The smart-cut process flow for demonstrating the GOI MIS photodetector. Thin films of 0.8–1.3 μm thick Ge were able to be transferred to the handle wafer.

The bulk Ge photodetector can detect the infrared at wavelengths of 1,310 and 1,550 nm, but the cost and the device speed are issues. Thin-film Ge photodetectors can potentially reduce the cost and increase the device speed. The thin film Ge is usually demonstrated on the Si substrates. The Ge-on-Si photodetectors can achieve low dark current densities of few mA/cm^2^ with p-i-n [57] and MSM [58] configurations. However, an isolating Ge film may be preferable for high speed operation. The Ge-on-insulator (GOI) MIS photodetectors have been demonstrated by the process of smart cut (Figure 19) [59]. Thin films of 0.8–1.3 μm thick-Ge were used as the active layers.

An n-type, Sb doped Ge substrate was firstly prepared as a “host” wafer. The H^+^ ions with a dose of 1–1.5 × 10^17^ cm^−2^ and the energy of 150–200 keV were then implanted into the host wafer to form a deep weakened region. On the other wafer, 50–80 nm of thermal SiO_2_ was grown on a Si wafer prepared as the “handle” wafer.

**Figure 20.**
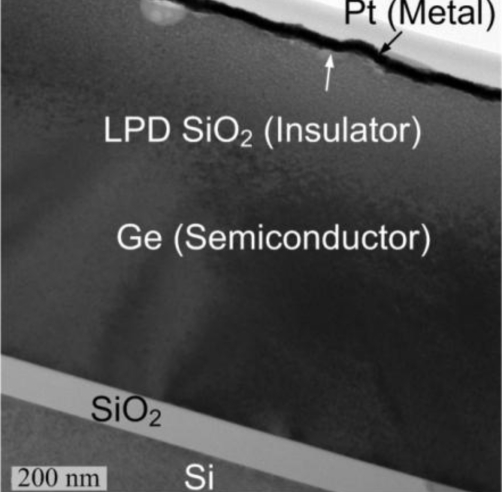
The TEM photograph of the 0.8 μm thick-Ge GOI MIS photodetector. The low-temperature LPD SiO_2_ was formed on the thin-film Ge instead of the unstable GeO_2_.

The handle and host wafers were cleaned hydrophilicly in SC1 (NH_4_OH + H_2_O_2_ + H_2_O) solution and the KOH aqueous solution, respectively. After rinsing in DI water and blown dry, the wafer pair was then initially bonded. Subsequently, the pair were annealed at 150 °C–300 °C for 1–24 hours to strengthen chemical bonds at the Ge/thermal-SiO_2_ interface and to induce thin-film transfer along the weakened hydrogen-ion-implanted region by H_2_ blistering. A ring-shaped Al was evaporated on the demonstrated GOI structure to form the Ohmic contact. The low-temperature LPD SiO_2_ and Pt gate electrode were used as the gate stack inside the Al ring contact to form the MIS diode. A TEM photograph of the GOI MIS photodetector is shown in Figure 20.

**Figure 21.**
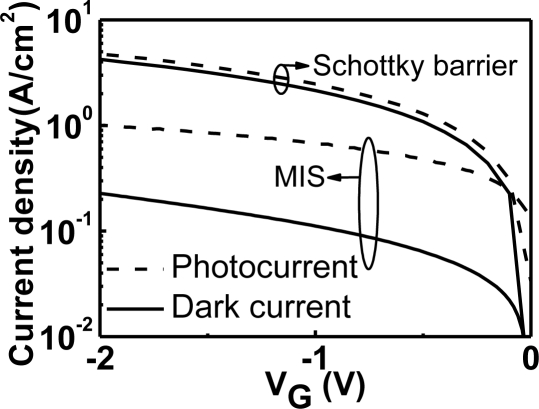
Dark currents and photocurrents of the 1.3 μm thick-Ge GOI MIS and SB photodetectors. As compared with the SB detector, the MIS detector had a smaller dark current and a larger responsivity.

In order to verify the advantages of the MIS structure, a GOI SB photodetector was also studied with a structure similar to the GOI MIS photodetector without the LPD SiO_2_ layer. At −2 V bias, the dark current density of the 1.3 μm thick-Ge GOI MIS detector was 0.23 A/cm^2^, while that of the GOI SB detector was 4.2 A/cm^2^ (Figure 21). Meanwhile, the MIS detector has the responsivity (at the 1,310 nm wavelength) of 230 mA/W larger than 160 mA/W of the SB detector.

For the SB diode, the dark current at negative bias (reverse bias) was dominated by the thermionic emission current of electrons from Pt to Ge, and the magnitude of the current was determined by the barrier height for electrons between Pt and Ge. However, for the MIS diode, the effective barrier height was large due to the insertion of a thin insulator layer. Note that the insulator layer was LPD SiO_2_, where defects at various levels were distributed. In insulator, if no energy level of traps, which assisted tunneling, was exactly the same with the conduction band edge of Ge at the LPD-SiO_2_/Ge interface, the effective barrier height was increased. Electrons should overcome the energy difference between the lowest traps (above the conduction band edge of Ge at the LPD-SiO_2_/Ge interface) and the Fermi level of Pt. In addition, when Pt directly contacted with Ge, the work function of Pt would be pinned from the un-contact level below the valence band edge of Ge to the gap state of Ge. An insulator layer between Pt and Ge could suppress the Fermi level pinning [60,61]. Hence, at negative bias, the thermionic emission current could be neglected for the MIS diode due to the large barrier height, and the dark current of the MIS diode was only dominated by the thermal generation current in Ge. Therefore, the MIS photodetector could reduce the dark current as compared with the SB photodetector.

Due to the Fermi level pinning of the SB diode, the depletion width of the SB diode was decreased. The MIS diode had a larger depletion width since the Fermi level pinning was suppressed and a deep depletion region was formed below the insulator layer. The electric field in the depletion region helped electron-hole pairs to be swept separately to form the photocurrent rather than be recombined via defects. The end result was the GOI MIS device displayed a higher responsivity compared with the GOI SB device.

**Figure 22.**
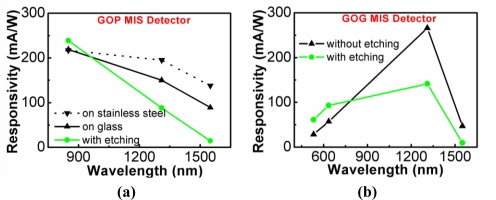
The responsivities of: **(a)** GOG MIS photodetectors; **(b)** GOP MIS photodetectors. Reprinted from [62,63] with permission of the American Institute of Physics (Copyright 2007 & 2009).

Using the smart-cut process technique, thin film Ge layers were also transferred onto a transparent glass substrate and a flexible polyimide substrate to form Ge-on-glass (GOG) [62] and Ge-on-polyimide (GOP) [63] structures. The responsivities of GOG and GOP MIS photodetectors are shown in Figure 22(a) and Figure 22(b), respectively. After the smart-cut processes, the surfaces of thin-film Ge were rough and defective due to the blistering of H_2_. These defective regions could be etched away by the chemical etching in the SC1 solution [64].

The dark currents and photocurrents under 532 nm illumination of unetched and etched GOG MIS photodetectors were studied. The dark current of the etched detector was lower than that of the unetched detector. With removal of the top defective region, the thermally generated carriers via the SiO_2_/Ge interface and the depletion region in Ge could be reduced. Hence, a lower dark current could be achieved for the etched detector. Photo-generated electron-hole pairs might recombine via defects rather than contribute to the photocurrents. Damage removal also increased the photocurrent under 532 nm illumination, which was mostly absorbed within the top thin layer of Ge. As compared with the unetched devices, the responsivities of etched devices were able to be enhanced as long as the remaining Ge was sufficiently thick for absorption. The drop of responsivities at 1,310 and 1,550 nm wavelengths after etching was due to the insufficient thickness of Ge layers. Such phenomena were both observed for the GOG MIS detector and GOP MIS detector (Figure 22).

The handle substrate of the GOP MIS photodetector was much thinner than that of the GOG MIS photodetector. The GOP MIS detector could be put on a reflective holder to improve the responsivities at longer wavelengths (Figure 22(b)). When the GOP MIS detector was on the reflective stainless steel, the unabsorbed incident light (at the 1,310 nm wavelength for example) was reflected from the reflective stainless steel and then reabsorbed in Ge. Therefore, the responsivity was enhanced. For the shorter wavelength at 850 nm, most of the incident light was absorbed before reflection, and the enhancement of the responsivity with the reflective holder was not observed.

## 5. MIS IR Photodetectors

Mid-wavelength (3–5 μm) and long-wavelength (8–14 μm) infrared photodetectors are attractive for environmental monitoring, military, astronomy, medicine and a myriad of many other applications. Well developed infrared photodetectors are composed of mercury cadmium telluride (HgCdTe or MCT). The HgCdTe material has advantages of both a high absorption coefficient and low thermal generation rate, which make it broadly used for infrared detection [65]. HgCdTe photodetectors based on MIS structures will be introduced in this section. Gradually, III–V compound semiconductor quantum well infrared photodetectors and quantum dot infrared photodetectors caused attention [66,67]. However, with the advantages of low cost and integration, high-performance Si-based quantum dot (well) photodetectors are more desirable. The MIS SiGe/Si quantum dot (well) infrared photodetectors will be discussed in this section, too.

### 5.1. HgCdTe MIS IR Photodetectors

**Figure 23.**
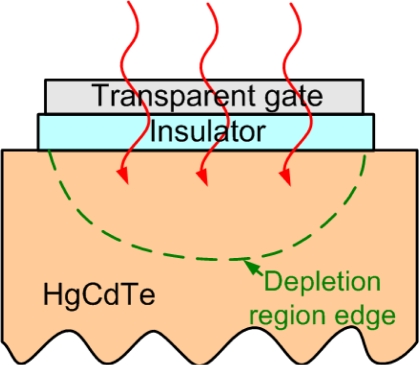
The typical structure of the HgCdTe MIS photodetectors. A pulse of voltage was applied to induce a deep-depletion region in the HgCdTe semiconductor for separation of photo-generated carriers.

HgCdTe photodetectors have been in existence for about five decades, and a variety of detector structures such as photoconductors or photodiodes were developed [68]. Among these, the HgCdTe photodiodes based on the n^+^-p and P^+^-n double-layer heterojunction structures are the most common. HgCdTe MIS photodetectors have attracted attention from ∼1970. The typical structure of HgCdTe MIS photodetectors is shown in Figure 23. Usually, a voltage pulse is applied to induce a deep-depletion region in the HgCdTe semiconductor for separation of photo-generated carriers. The common insulator formation of HgCdTe MIS structures was classified into two categories [69]. One was the native oxide films. The other was the deposited films, including ZnS, SiO_2_, and CdTe *etc.* The advantage of the MIS structure was that the detection device and the readout circuit could be well integrated. However, the typical pulse operation resulted in some shortcomings. An additional dark current was formed due to the trap-assisted tunneling in the deep-depletion region in the semiconductor. The near defect-free active layer was needed to suppress such a dark current, but it was difficult to obtain. Hence, most of the investigation effort on the HgCdTe MIS photodetectors was abandoned ∼1987 [68,70].

Some subsequent research focused on the investigation of passivation in the HgCdTe MIS structures since the effective passivation of surface states could reduce the dark currents as well. For example, passivation by CdZnTe was investigated by Lee *et al.* [71], and ammonium sulfide was used to improve the interface of common metal/ZnS/HgCdTe MIS structures [72]. In [71], bromine etching or intentional oxidation of HgCdTe was performed before the deposition of CdZnTe on HgCdTe, to investigate the corresponding properties of passivation. Fixed charges, slow and fast interface states were desired to be minimized to avoid the degradation of the HgCdTe detector performance. The etching by a 1% bromine in the methanol solution should be shorter than one minute to minimize the fixed charge. Three intentional oxidation processes including air-exposure oxidation, anodic oxidation, and photochemical oxidation were compared. Air-exposure oxidation resulted in smaller slow interface states as compared with the anodic oxidation. The C-V curve of the photochemical-oxidation sample showed the largest stretch-out, which was caused by a large amount of dangling bonds acting as the fast interface states.

Jung *et al.* showed that the fixed charge density of 3.2 × 10^11^ cm^−2^ of the sulfide treated MIS sample was smaller than that of 6.4 × 10^11^ cm^−2^ of the untreated sample in [72]. The slow state density also decreased from 5.5 × 10^11^ cm^−2^ to 7.5 × 10^10^ cm^−2^ after sulfidation. Such improvement was due to the suppression of native oxidation on the surface with the sulfidation process.

Different passivation insulator layers were also used to suppress Fermi level pinning of HgCdTe MIS photodetectors [73]. These insulator layers included the native oxide, Al_2_O_3_, and SiO_2_ layers. The native oxide layers were formed on an insulated holder outside the plasma, and the ion bombarding was avoided. The fixed charge density and interface trap density were as low as 1.5 × 10^11^ cm^−2^ and 3 × 10^11^ cm^−2^ eV^−1^. respectively. Al_2_O_3_ and SiO_2_ layers were formed by RF magnetron sputtering with 0.011 Pa of oxygen in an argon atmosphere. Their fixed charge densities and interface trap densities were smaller than 6.0 × 10^10^ cm^−2^ and 5 × 10^11^ cm^−2^ eV^−1^. The interface quality is high enough to eliminate the Fermi level pinning. It could be expected that continuous investigation on HgCdTe photodetectors based on the MIS structure will be performed. However, it will be a difficult assignment for the HgCdTe MIS photodetectors to compete with the HgCdTe pn-junction-type photodetectors.

### 5.2. MIS SiGe/Si Quantum Dot (Well) IR Photodetectors

**Figure 24.**
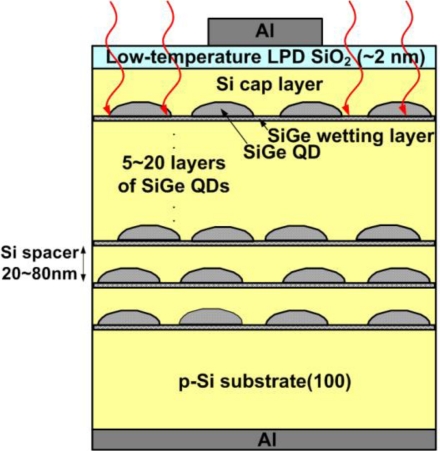
The schematic structure of MIS SiGe/Si QDIPs. The low-temperature LPD SiO_2_ layer was adopted in order to avoid the strain relaxation and interdiffusion between Si and Ge.

Due to the natural valence band offset at the SiGe/Si heterojunction, discrete energy levels could be formed inside the Si/SiGe/Si quantum confinement region. The transition between these discrete levels can be used for infrared detection. The SiGe/Si quantum dots and quantum wells were broadly investigated for mid-infrared and long-wavelength detection [74–76]. The SiGe/Si quantum-dot structure has been fabricated into MIS quantum dot infrared photodetectors (QDIPs), too [77]. The SiGe quantum dots were formed on the Si substrate by the Stranski-Krastanov (SK) mode [78]. The insulator layer was then deposited on the semiconductor substrate by the low-temperature LPD process in order to avoid the strain relaxation and interdiffusion between Si and Ge. The schematic structure of MIS SiGe/Si QDIPs is shown in Figure 24.

It is worth mentioning that the suitable gate electrode selected here is Al instead of Pt, which has been suggested in Figure 18. In Figure 18, the semiconductor is n-type, and the selection of the high work-function metal, Pt, can suppress the electron tunneling current from the metal to the semiconductor at the inversion (negative) bias. However, for this SiGe/Si QDIP structure, the semiconductor is p-type. Pt will lead to a hole tunneling current at the inversion (positive) bias [79], which should be avoided.

**Figure 25.**
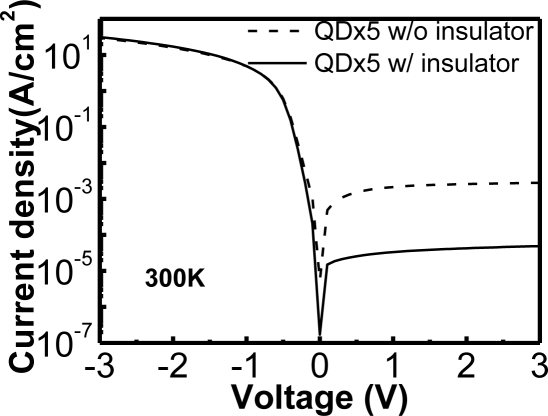
Dark currents of five-period SiGe/Si QDIPs with and without an insulator layer between the metal and semiconductor. A QD sample with the insulator layer indeed can reduce the dark inversion current. Reproduced from [80] with permission of Elsevier (2006).

The J-V curves of SiGe/Si QDIPs with and without the insertion of an insulator layer between the metal and semiconductor are compared in Figure 25 [80]. The insertion of an insulator layer to fabricate the MIS photodetector could indeed reduce the dark current. The mechanism of the dark current reduction of the MIS structure was similar to that discussed in the Subsection 4.3. For the MIS SiGe/Si QDIP, the thermionic emission current of holes from Al to the semiconductor was able to be significantly reduced due to the extra barrier and the suppression of Fermi level pinning with an insulator layer.

**Figure 26.**
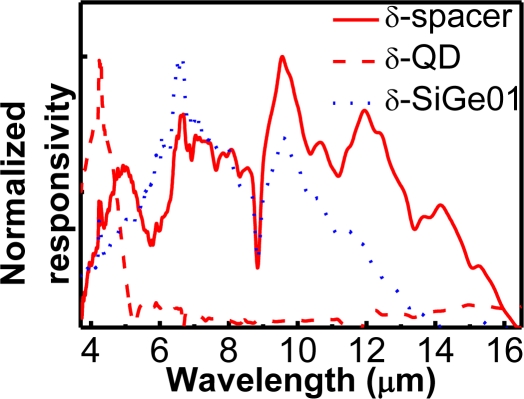
Spectral responses of the MIS SiGe/Si quantum dot (well) infrared photodetectors at 15 K. For the spacer sample, the detection regions of 3.7–6 and 6–16 μm corresponded to the LH1-to-LH3 transition in QDs, and intraband transitions in the boron doping wells, respectively [83].

Spectral responses of MIS SiGe/Si quantum dot (well) infrared photodetectors are shown in Figure 26 [81]. The label “δ-spacer” and “δ-QD” indicated the MIS SiGe/Si QDIPs with boron doping in the Si spacers and SiGe QDs, respectively. The label “δ-SiGe01” indicated the MIS SiGe/Si quantum well infrared photodetector with boron doping in the Si_0.9_Ge_0.1_ quantum wells. For the spacer sample, the detection regions of 3.7–6 and 6–16 μm corresponded to the LH1-to-LH3 transition in SiGe/Si QDs, and intraband transitions in boron doping wells, respectively.

For the SiGe01 sample, since the depth of Si_0.9_Ge_0.1_ quantum wells would be very shallow unlike that of SiGe QDs (Ge composition of ∼0.5 [82]), the LH1-to-LH3 transition would not exist. Only the intraband transitions in the boron doping wells contributed to the detection spectrum. Due to the additional quantum confinement of the SiGe QW outside the boron doping well, the detection spectrum had a slight blue shift as compared with the spacer sample. For the QD sample, the excited holes via intraband transitions in the boron doping wells were subsequently blocked by the large valence band barrier of the SiGe/Si QDs. Hence, detection via intraband transitions in the boron doping wells was not observed, and only the narrow spectrum due to the LH1-to-LH3 transition in SiGe QDs was observed. Although the spectrum of the QD sample was narrower than that of the spacer sample, a lower dark current and larger detectivity was able to be achieved with such a structure [83].

## 6. Conclusions

Nowadays, integrated circuits are composed of MOSFETs. If the MIS structure can be utilized for photodetectors, the integration of detection devices and readout circuit will be much easier and less expensive to manufacture. The wide-bandgap semiconductor has been utilized in the MIS UV photodetectors, and the optimized UV-to-visible rejection ratio could be more than three orders of magnitude. Si-based and thin-film Ge MIS photodetectors are suitable for visible-to-near-IR photo-detection. With the MIS SiGe/Si QDIPs, a broadband infrared detection covering atmospheric transmission windows at 3–5.3 μm and 7.5–14 μm is possible.

## Figures and Tables

**Table 1.** Characteristics of III–V nitride based UV Photodetectors with different configurations.

**Device**	***J_rev_* (*J_inv_*) (A/cm^2^)**	**Responsivity (mA/W)**	**Detectivity (cmHz^0.5^W^−1^)**	**Rejection ratio**	**Ref.**
MIS ITO/photo-SiO_2_/GaN	5 × 10^−7^ @1 V	110@338 nm@3 V	2 × 10^8^@16 V	∼200@3 V	[19]
MIS Al/LPD-SiO_2_/GaN	7 × 10^−7^ @1 V	112@366 nm@10 V			[21]
p-i-n GaN	6 × 10^−8^@1 V	150@363 nm@0 V		10^6^@0 V	[24]
p-i-n GaN	4 × 10^−11^@10 V	140@360 nm@20 V	1.7 × 10^14^@20 V	8×10^3^@20 V	[25]
MSM GaN	4.5 × 10^−6^@10 V	300@350 nm@6 V		∼200@6 V	[26]
MSM GaN	10^−6^@1 V	192@361 nm@3 V	6.4 × 10^9^@3 V	∼10^3^@3 V	[27]

MIS AlGaN/GaN	∼10^−6^ @1 V	144@350 nm@5 V		3,000@5 V	[23]
p-i-n AlGaN/GaN	6 × 10^−10^ @1 V	200@355 nm@0 V		10^4^@0 V	[28]
p-i-n AlGaN/GaN	3 × 10^−11^ @6 V	65@267 nm@0 V	4.9 × 10^14^@0 V	∼10^4^@0 V	[29]
MSM AlGaN/GaN	∼10^−6^ @6 V	2@264 nm@0 V	8.9 × 10^10^@0 V	2 × 10^4^@0 V	[29]
MSM AlGaN/GaN	∼10^−3^ @1 V	2@350 nm@5 V		200@5 V	[23]

## References

[1] Monroy E, Omnès F, Calle F (2003). Wide-Bandgap Semiconductor Ultraviolet Photodetectors. Semicond. Sci. Technol.

[2] Park JS, Lin TL, Jones EW, Castillo HMD, Gunapala SD (1994). Long-Wavelength Stacked SiGe/Si Heterojunction Internal Photoemission Infrared Detectors Using Multiple SiGe/Si Layers. Appl. Phys. Lett.

[3] Rogalski A (2003). Quantum Well Photoconductors in Infrared Detector Technology. J. Appl. Phys.

[4] Kasper E, Oehme M (2008). High Speed Germanium Detectors on Si. Phys. Stat. Sol. C.

[5] Brennan KF, Haralson J, Parks JW, Salem A (1999). Review of Reliability Issues of Metal-Semiconductor-Metal and Avalanche Photodiode Photonic Detectors. Microelectron. Reliab.

[6] Lee HC, Su YK, Lin JC, Cheng YC, Wu SL, Jhou YD (2009). AlInGaN Metal-Insulator-Semiconductor Photodetectors at UV-C 280 nm. Electrochem. Solid. St.

[7] Lo SH, Buchanan DA, Taur Y, Wang W (1997). Quantum-Mechanical Modeling of Electron Tunneling Current from the Inversion Layer of Ultra-Thin-Oxide MOSFET’s. IEEE. Electron. Device. Lett.

[8] Lee WC, Hu C (2001). Modeling CMOS Tunneling Currents Through Ultrathin Gate Oxide Due to Conduction and Valence Band Electron and Hole Tunneling. IEEE. Trans. Electron. Devices.

[9] Liu CW, Liu WT, Lee MH, Kuo WS, Hsu BC (2000). A Novel Photodetector Using MOS Tunneling Structures. IEEE. Electron. Device. Lett.

[10] Lin CH, Hsu BC, Lee MH, Liu CW (2001). A Comprehensive Study of Inversion Current in MOS Tunneling Diodes. IEEE. Trans. Electron. Devices.

[11] Sze SM, Ng KK (2007). Physics of Semiconductor Devices.

[12] Monroy E, Calle F, Pau JL, Muñoz E, Omnès F, Beaumont B, Gibart P (2001). Application and Performance of GaN Based UV Detectors. Phys. Stat. Sol. A.

[13] Sze SM, Ng KK (2007). Physics of Semiconductor Devices.

[14] Liu CY, Chen BY, Tseng TY (2004). Deep Depletion Phenomenon of SrTiO_3_ Gate Dielectric Capacitor. J. Appl. Phys.

[15] Liu J, Michel J, Giziewicz W, Pan D, Wada K, Cannon DD, Jongthammanurak S, Danielson DT, Kimerling LC (2005). High-Performance, Tensile-Strained Ge p-i-n Photodetectors on a Si Platform. Appl Phys Lett.

[16] Monroy E, Calle F, Pau JL, Muñoz E, Omnès F (2000). Low-Noise Metal-Insulator-Semiconductor UV Photodiodes Based on GaN. Electron. Lett.

[17] Basak D, Amin G, Mallik B, Paul GK, Sen SK (2003). Photoconductive UV Detectors on Sol-Gel-Synthesized ZnO Films. J. Cryst. Growth.

[18] Muñoz E, Monroy E, Pau JL, Calle F, Omnès F, Gibart P (2001). Nitrides and UV Detection. J. Phys. Condens. Matter.

[19] Chiou YZ, Su YK, Chang SJ, Gong J, Chang CS, Liu SH (2002). The Properties of Photo Chemical-Vapor Deposition SiO_2_ and its Application in GaN Metal-Insulator Semiconductor Ultraviolet Photodetectors. J. Electron. Mater.

[20] Lin CH, Yu CY, Peng CY, Ho WS, Liu CW (2007). Broadband SiGe/Si Quantum Dot Infrared Photodetectors. J Appl Phys.

[21] Hwang JD, Yang GH, Yang YY, Yao PC (2005). Nitride-Based UV Metal-Insulator-Semiconductor Photodetector with Liquid-Phase-Deposition Oxide. Jpn. J. Appl. Phys.

[22] Walker D, Kumar V, Mi K, Sandvik P, Kung P, Zhang XH, Razeghi M (2000). Solar-Blind AlGaN Photodiodes with Very Low Cutoff Wavelength. Appl. Phys. Lett.

[23] Chang PC, Chen CH, Chang SJ, Su YK, Yu CL, Huang BR, Chen PC (2006). High UV/visible Rejection Contrast AlGaN/GaN MIS Photodetectors. Thin Solid Films.

[24] Walker D, Saxler A, Kung P, Zhang X, Hamilton M, Diaz J, Razeghi M (1998). Visible Blind GaN p-i-n Photodiodes. Appl. Phys. Lett.

[25] Zhang Y, Shen SC, Kim HJ, Choi S, Ryou JH, Dupuis RD, Narayan B (2009). Low-Noise GaN Ultraviolet p-i-n Photodiodes on GaN Substrates. Appl Phys Lett.

[26] Carrano JC, Grudowski PA, Eiting CJ, Dupuis RD, Campbell JC (1997). Very Low Dark Current Metal-Semiconductor-Metal Ultraviolet Photodetectors Fabricated on Single-Crystal GaN Epitaxial Layers. Appl. Phys. Lett.

[27] Wang CK, Chang SJ, Su YK, Chiou YZ, Chen SC, Chang CS, Lin TK, Liu HL, Tang JJ (2006). GaN MSM UV Photodetectors with Titanium Tungsten Transparent Electrodes. IEEE. Trans. Electron. Devices.

[28] Yang W, Nohova T, Krishnankutty S, Torreano R, McPherson S, Marsh H (1998). Back-Illuminated GaN/AlGaN Heterojunction Photodiodes with High Quantum Efficiency and Low Noise. Appl. Phys. Lett.

[29] Ozbay E, Biyikli N, Kimukin I, Kartaloglu T, Tut T, Aytür O (2004). High-Performance Solar-Blind Photodetectors Based on Al_x_Ga_1-x_N Heterostructures. IEEE. J. Sel. Top. Quantum Electron.

[30] Razeghi M, Rogalski A (1996). Semiconductor Ultraviolet Detectors. J. Appl. Phys.

[31] Liu CH, Chang CS, Chang SJ, Su YK, Chiou YZ, Liu SH, Huang BR (2003). The Characteristics of Photo-CVD SiO_2_ and Its Application on SiC MIS UV Photodetectors. Mater. Sci. Eng. B.

[32] Chen X, Zhu H, Cai J, Wu Z (2007). High-Performance 4H-SiC-Based Ultraviolet p-i-n Photodetector. J Appl Phys.

[33] Morkoc H, Strite S, Gao GB, Lin ME, Sverdlov B, Burns M (1994). Large-Band-Gap SiC, III–V Nitride, and II–VI ZnSe-Based Semiconductor Device Technologies. J. Appl. Phys.

[34] Lin TK, Chang SJ, Chiou YZ, Wang CK, Chang SP, Lam KT, Sun YS, Huang BR (2006). Homoepitaxial ZnSe MIS Photodetectors with SiO_2_ and BST Insulator Layers. Solid. State. Electron.

[35] Young SJ, Ji LW, Chang SJ, Liang SH, Lam KT, Fang TH, Chen KJ, Du XL, Xue QK (2008). ZnO-Based MIS Photodetectors. Sens. Actuat. A. Phys.

[36] Tsukazaki A, Ohtomo A, Onuma T, Ohtani M, Makino T, Sumiya M, Ohtani K, Chichibu SF, Fuke S, Segawa Y, Ohno H, Koinuma H, Kawasaki M (2005). Repeated Temperature Modulation Epitaxy for p-Type Doping and Light-Emitting Diode Based on ZnO. Nature Mater.

[37] Zhu H, Shan CX, Wang LK, Zheng J, Zhang JY, Yao B, Shen DZ (2010). Metal-Oxide-Semiconductor-Structured MgZnO Ultraviolet Photodetector with High Internal Gain. J. Phys. Chem. C.

[38] Yang W, Vispute RD, Choopun S, Sharma RP, Venkatesan T, Shen H (2001). Ultraviolet Photoconductive Detector Based on Epitaxial Mg_0.34_Zn_0.66_O Thin Films. Appl. Phys. Lett.

[39] Ju ZG, Shan CX, Jiang DY, Zhang JY, Yao B, Zhao DX, Shen DZ, Fan XW (2008). Mg_x_Zn_1-x_O-Based Photodetectors Covering the Whole Solar-Blind Spectrum Range. Appl Phys Lett.

[40] Ho WS, Lin CH, Cheng TH, Hsu WW, Chen YY, Kuo PS, Liu CW (2009). Narrow-Band Metal-Oxide-Semiconductor Photodetector. Appl Phys Lett.

[41] Hansen TE (1978). Silicon UV-Photodiodes Using Natural Inversion Layers. Phys. Scr.

[42] Colace L, Assanto G, Fulgoni D, Nash L (2008). Near-Infrared p-i-n Ge-on-Si Photodiodes for Silicon Integrated Receivers. J. Lightwave. Technol.

[43] Sher A, Tsuo YH, Moriarty JA, Miller WE, Crouch RK (1980). Si and GaAs Photocapacitive MIS Infrared Detectors. J. Appl. Phys.

[44] Malik A, Grimalsky V (2004). Conception of the Optical Sensors Based on Transient Processed in MIS Structures. Sens. Actuat. A. Phys.

[45] Malik A, Grimalsky V, Jacome AT, Durini D (2004). Theoretical Modeling and Experimental Investigation of MIS Radiation Sensor with Giant Internal Signal Amplification. Sens. Actuat. A. Phys.

[46] Thaniyavarn S, Gustafson TK (1982). Metal/Tunnel-Barrier/Semiconductor/Tunnel-Barrier/Metal Fast Photodetector. Appl. Phys. Lett.

[47] Fernandes M, Vygranenko Y, Schwarz R, Vieira M (2001). ITO/SiO*_x_*/Si Optical Sensor with Internal Gain. Sens. Actuat. A. Phys.

[48] Hsu BC, Chang ST, Shie CR, Lai CC, Chen PS, Liu CW (2002). High Efficient 820 nm MOS Ge Quantum Dot Photodetectors for Short-Reach Integrated Optical Receivers with 1,300 and 1,550 nm Sensitivity. IEDM Tech Dig.

[49] Cheng JY, Lu HT, Hwu JG (2010). Metal-Oxide-Semiconductor Tunneling Photodiodes with Enhanced Deep Depletion at Edge by High-*k* Material. Appl Phys Lett.

[50] Hsu BC, Liu CW, Liu WT, Lin CH (2001). A PMOS Tunneling Photodetector. IEEE. Trans. Electron. Devices.

[51] Hashimoto H, Yamada R, Hirokane T, Arima K, Uchikoshi J, Morita M (2007). Photodetective Characteristics of Metal-Oxide-Semiconductor Tunneling Structure with Aluminum Grid Gate. Jpn. J. Appl. Phys.

[52] Seto M, Rochefort C, Jager SD, Hendriks RFM, ’t Hooft GW, Mark MBVD (1999). Low-Leakage-Current Metal-Insulator-Semiconductor-Insulator-Metal Photodetector on Silicon with a SiO_2_ Barrier-Enhancement Layer. Appl. Phys. Lett.

[53] Hsu BC, Chang ST, Chen TC, Kuo PS, Chen PS, Pei Z, Liu CW (2003). A High Efficient 820 nm MOS Ge Quantum Dot Photodetector. IEEE. Electron. Device. Lett.

[54] Binari SC, Miller WE, Sher A, Tsuo YH (1979). Ge Photocapacitive MIS Infrared Detectors. J. Appl. Phys.

[55] Cheng TH, Liao MH, Yeh L, Lee TL, Liang MS (2008). Digital Communication Using Ge Metal-Insulator-Semiconductor Light-Emitting Diodes and Photodetectors. J Appl Phys.

[56] Kuo PS, Fu YC, Chang CC, Lee CH, Liu CW (2007). Dark Current Reduction of Ge MOS Photodetectors by High Work Function Electrodes. Electron. Lett.

[57] Morse M, Dosunmu O, Sarid G, Chetrit Y (2006). Performance of Ge-on-Si p-i-n Photodetectors for Standard Receiver Modules. IEEE. Photon. Technol. Lett.

[58] Zang H, Lee SJ, Loh WY, Wang J, Yu MB, Lo GQ, Kwong DL, Cho BJ (2008). Application of Dopant Segregation to Metal-Germanium-Metal Photodetectors and Its Dark Current Suppression Mechanism. Appl Phys Lett.

[59] Yu CY, Lee CY, Lin CH, Liu CW (2006). Low-Temperature Fabrication and Characterization of Ge-on-Insulator Structures. Appl Phys Lett.

[60] Nishimura T, Kita K, Toriumi A (2007). Evidence for Strong Fermi-Level Pinning Due to Metal-Induced Gap States at Metal/Germanium Interface. Appl Phys Lett.

[61] Lieten RR, Degroote S, Kuijk M, Borghs G (2008). Ohmic Contact Formation on n-Type Ge. Appl Phys Lett.

[62] Lin CH, Chiang YT, Hsu CC, Lee CH, Huang CF, Lai CH, Cheng TH, Liu CW (2007). Ge-on-Glass Detectors. Appl Phys Lett.

[63] Ho WS, Dai YH, Deng Y, Lin CH, Chen YY, Lee CH, Liu CW (2009). Flexible Ge-on-Polyimide Detectors. Appl Phys Lett.

[64] Heyns M, Meuris M, Caymax M (2006). Ge and III/V as Enabling Materials for Future CMOS Technologies. ECS Trans.

[65] Rogalski A (2003). Infrared Detectors: Status and Trends. Prog. Quantum. Electron.

[66] Deng SY, Lee JYM, Lai JT, Chih YD, Sun TP, Hong HM (1995). Front-Illuminated Long Wavelength Multiple Quantum-Well Infrared Photodetectors with Backside Gratings. J. Appl. Phys.

[67] Finkman E, Maimon S, Immer V, Bahir G, Schacham SE, Fossard F, Julien FH, Brault J, Gendry M (2001). Polarized Front-Illumination Response in Intraband Quantum Dot Infrared Photodetectors at 77 K. Phys Rev B.

[68] Rogalski A (2005). HgCdTe Infrared Detector Material: History, Status and Outlook. Rep. Prog. Phys.

[69] Singh R, Gupta AK, Chhabra KC (1991). Surface Passivation of Mercury-Cadmium-Telluride Infrared Detectors. Def. Sci. J.

[70] Kinch MA, Capper P (1994). MIS Devices in HgCdTe. Properties of Narrow Gap Cadmium-Based Compounds (EMIS Datareviews Series No 10).

[71] Lee TS, Choi KK, Jeoung YT, Kim HK, Kim JM, Kim YH, Chang JM, Song WS, Kim SU, Park MJ, Lee SD (1997). Surface Passivation of HgCdTe by CdZnTe and Its Characteristics. J. Electron. Mater.

[72] Jung YC, An SY, Suh SH, Choi DK, Kim JS (2005). Ammonium Sulfide Treatment of HgCdTe Substrate and Its Effects on Electrical Properties of ZnS/HgCdTe Heterostructure. Thin Solid Films.

[73] Damnjanoviè V, Ponomarenko VP, Elazar JM (2009). Photo-Electric Characteristics of HgCdTe Tunnel MIS Photo-Detectors. Semicond Sci Technol.

[74] Karunasiri RPG, Park JS, Wang KL (1991). Si_1-x_Ge_x_/Si Multiple Quantum Well Infrared Detector. Appl. Phys. Lett.

[75] Kruck P, Helm M, Fromherz T, Bauer G, Nützel JF, Abstreiter G (1996). Medium-Wavelength, Normal-Incidence, p-Type Si/SiGe Quantum Well Infrared Photodetector with Background Limited Performance up to 85 K. Appl. Phys. Lett.

[76] Tong S, Liu F, Khitun A, Wang KL, Liu JL (2004). Tunable Normal Incidence Ge Quantum Dot Midinfrared Detectors. J. Appl. Phys.

[77] Hsu BC, Lin CH, Kuo PS, Chang ST, Chen PS, Liu CW, Lu JH, Kuan CH (2004). Novel MIS Ge-Si Quantum-Dot Infrared Photodetectors. IEEE. Electron. Device. Lett.

[78] Liu JL, Wu WG, Balandin A, Jin GL, Wang KL (1992). Intersubband Absorption in Boron-Doped Multiple Ge Quantum Dots. Appl. Phys. Lett.

[79] Kuo PS, Lin CH, Peng CY, Fu YC, Liu CW (2007). Transport Mechanism of SiGe Dot MOS Tunneling Diodes. IEEE. Electron. Device. Lett.

[80] Lin CH, Yu CY, Kuo PS, Chang CC, Guo TH, Liu CW (2006). Doped MOS Ge/Si Quantum Dot/Well Infrared Photodetector. Thin Solid Films.

[81] Lin CH, Yu CY, Chang CC, Lee CH, Yang YJ, Ho WS, Chen YY, Liao MH, Cho CT, Peng CY, Liu CW (2008). SiGe/Si Quantum-Dot Infrared Photodetectors with Doping. IEEE Trans. Nanotech.

[82] Liao MH, Lee CH, Hung TA, Liu CW (2007). The Intermixing and Strain Effects on Electroluminescence of SiGe Dots. J Appl Phys.

[83] Lin CH, Liu CW (2010). Metal-Oxide-Semiconductor SiGe/Si Quantum Dot Infrared Photodetectors with Delta Doping in Different Positions. Thin Solid Films.

